# Multidrug-Resistant *Escherichia coli* Harboring ESBL and MCR-1 Genes in Raw Milk Cheese and Chicken Carcasses in Egypt: Characterization and Control Using Algal Extracts

**DOI:** 10.3390/foods15183324

**Published:** 2026-09-19

**Authors:** Mustafa Sadek, Noura F. Mostafa, Eman Ezzat, Aya Seleem, Hani Saber, Waleed Younis, Sally S. Sakr, Asmahan A. Ali, Basma Gamal

**Affiliations:** 1 Department of Food Hygiene and Control (Meat Hygiene and Control), Faculty of Veterinary Medicine, Qena University, Qena 83523, Egypt; 2 Faculty of Veterinary Medicine, King Salman International University, South Sinai, Ras Sudr 46512, Egypt; 3 Gastrointestinal Surgery Center (GISC), Faculty of Medicine, Mansoura University, Mansoura 35516, Egypt; 4 Department of Zoonoses, Faculty of Veterinary Medicine, Cairo University, Cairo 12211, Egypt; 5 Department of Botany and Microbiology, Faculty of Science, Qena University, Qena 83523, Egypt; 6 Department of Microbiology, Faculty of Veterinary Medicine, Qena University, Qena 83523, Egypt; 7 Department of Food Science and Human Nutrition, College of Agriculture and Food, Qassim University, Buraydah 51452, Saudi Arabia; 8 Department of Food Hygiene and Control (Milk Hygiene and Control), Faculty of Veterinary Medicine, Qena University, Qena 83523, Egypt

**Keywords:** food safety, *Escherichia coli*, virulence, MCR, ESBLs, algal extracts, antimicrobial resistance, food supply

## Abstract

**Objectives:** This study investigates the occurrence and characteristics of extended-spectrum β-lactamase (ESBL)-producing and polymyxin-resistant *Escherichia coli* isolated from raw milk cheese and chicken carcasses in Egypt. In addition, the study evaluates the antibacterial activity of various algal extracts from *Padina pavonica* and *Polycladia myrica*, collected from the Red Sea, Egypt, against selected virulent and multidrug-resistant *E. coli* isolates. **Methods:** A total of 200 samples, comprising raw milk cheese samples (n = 100) and chicken carcass samples (n = 100), were examined for the presence of *E. coli*. Antimicrobial susceptibility testing was performed for all isolates using the disk diffusion and broth microdilution techniques. Phenotypic confirmation of various resistance traits was conducted to confirm resistance patterns. PCR screening was performed for various resistance genes and virulence-associated genes. Phylogenetic grouping analysis of the *E. coli* isolates examined was also performed. Two algal extracts of *P. pavonica* and *P. myrica* were screened using gas chromatography–mass spectrometry and evaluated against our panel of virulent MDR foodborne *E. coli* isolates using the resazurin-based microtiter dilution method. **Results:** Among the 200 food samples, *E. coli* was recovered from 14% of samples and confirmed by using *phoA* gene detection. Of the recovered isolates, 64.3% were multidrug-resistant (MDR), with the majority originating from chicken carcasses (88.9%). Most isolates (92.9%) carried ≥3 virulence genes, with *iss* (100%), *iutA* (92.9%), and *eaeA* (89.3%) being the predominant determinants. Potential EPEC profiles were the most frequently detected (60.7%), followed by EHEC profiles (28.6%), whereas potential STEC/non-EHEC hybrid and ExPEC profiles were detected in 7.1% and 3.6% of isolates, respectively. The *bla*_CTX-M_ gene predominated among ESBL determinants (96.4%), while *bla*_SHV_, *bla*_CMY-2_, and *mcr-1* were also detected. Notably, *mcr-1* was identified in 82.1% of isolates, with frequent co-occurrence of *mcr-1* and *ESBL* genes. Phylogenetic analysis revealed that most isolates belonged to group B1 (85.7%), whereas groups D and B2 accounted for 10.7% and 3.5%, respectively. In vitro antibacterial assays demonstrated that the *P. pavonica* methanol and ethyl acetate extracts and the *P. myrica* ethyl acetate extract exhibited inhibitory activity against MDR *E. coli*, with minimum inhibitory concentrations (MICs) ranging from 2.5 to 10 mg/mL. Overall, the *P. myrica* extract showed stronger antibacterial activity (MICs, 2.5–5 mg/mL) than the *P. pavonica* extracts (MICs, 5–10 mg/mL), highlighting their potential as natural antibacterial agents against MDR *E. coli*. **Conclusions:** These findings raise important public health concerns, as contaminated food products can act as reservoirs for the dissemination of multidrug-resistant (MDR) *E coli*, thereby increasing the risk of human infections ranging from mild gastrointestinal illnesses to severe, potentially life-threatening diseases. In addition, the present findings demonstrate the promising antibacterial activity of methanolic and ethyl acetate extracts of *P. myrica* and *P. pavonica* against MDR *E. coli* isolates, supporting their potential as sources of natural antibacterial compounds and warranting further investigation of their efficacy, mechanisms of action, and active constituents.

## 1. Introduction

*Escherichia coli* is a well-known enterobacterial species and a commensal in humans and animals that can be transmitted from animals to humans directly via the food chain and/or the environment, causing a diversity of diseases in humans such as diarrhea outbreaks, gastroenteritis, and foodborne illness, particularly in developing countries [1,2]. The pathogenicity of *E. coli* is driven by its virulence genes, which enable host colonization, nutrient acquisition, and immune evasion [3]. Based on their virulence characteristics, diarrheagenic *E. coli* (DEC) strains are classified into eight major pathotypes, including enteropathogenic *E. coli* (EPEC), Shiga toxin-producing *E. coli* (STEC), and enterohemorrhagic *E. coli* (EHEC) [4]. The detection of key virulence genes provides a reliable approach for differentiating *E. coli* pathotypes. In particular, the presence of *stx1* and/or *stx2* identifies STEC, whereas the combination of *stx1* and/or *stx2* with the *eae* gene, which encodes the adhesion protein intimin, is indicative of the EHEC pathotype [5].

The contamination of food of animal origin with a virulent *E. coli* strain poses a significant threat to consumers’ health. STEC is considered the most concerning *E. coli* pathotype in food safety. In 2024, a comprehensive review documented over 39,787 STEC cases associated with 1343 outbreaks since 1982, primarily linked to food consumption [6]. STEC infection may lead to severe and potentially fatal illnesses in humans, including bloody diarrhea, hemorrhagic colitis, and hemolytic uremic syndrome, leading to acute renal failure [7]. The pathogenicity of STEC is mainly reliant on the production of one or two Shiga toxins (Stx1 and Stx2). Foodborne STEC outbreaks have been frequently associated with the consumption of undercooked ground beef, raw milk, and raw milk cheeses, highlighting the importance of surveillance along the food production chain [8]. 

*E. coli* exhibits a high capacity to acquire and accumulate antimicrobial resistance genes, leading to phenotypic resistance against antibiotics traditionally used for treatment. These resistance genes can be transmitted to other bacteria, including commensals, through horizontal gene transfer via mobile elements such as plasmids, transposons, and integrons, facilitating their spread and dissemination [9].

The dramatic and uncontrolled increase in antibiotic resistance, particularly to β-lactam antibiotics, has become a serious global public health issue globally. One of the most important mechanisms of β-lactam resistance is the production of extended-spectrum β-lactamases (ESBLs) [10]. ESBLs, particularly *bla*_CTX-M_-like, are mainly produced by Enterobacterales, including *E. coli*, and are able to hydrolyze a wide range of β-lactam antibiotics, including broad-spectrum cephalosporins and monobactams [11]. Several studies have reported the presence of ESBL-producing *E. coli* in many types of food, such as meat, milk and various types of cheese [12,13,14,15].

Colistin, a polymyxin antibiotic, is regarded as one of the last-resort therapeutic options for treating infections caused by MDR Gram-negative bacteria. However, the emergence of plasmid-mediated colistin resistance genes (mcr), particularly mcr-1, has raised serious global public health concerns due to their potential for rapid horizontal dissemination [16]. The widespread and uncontrolled use of colistin in veterinary practice, including its use as a growth promoter in livestock, has accelerated the emergence and spread of colistin-resistant *E. coli*, particularly in food-producing animals and food products [17,18,19,20]. In 2011, Egypt officially banned the use of colistin for veterinary purposes in food animals [21]. However, recent studies indicate that the ban has not been fully enforced, as colistin continues to be used in livestock, mostly for non-therapeutic purposes, and is frequently included in routine management protocols for poultry from day one [22].

The current study was conducted to determine the contamination rates of commonly consumed food items in Egypt, raw milk cheeses and chicken carcasses, with such virulent *E. coli* strains, and to characterize the extended-spectrum ß-lactamase (ESBL) producing and polymyxin-resistant *E. coli* strains recovered from these food samples. Raw milk cheese and chicken carcasses, a common source of animal protein in the Egyptian diet, are widely consumed in Egypt and represent significant potential vehicles for foodborne pathogens. This study also evaluates the antibacterial activity of various algal crude extracts from two brown algal species, *Padina pavonica* and *Polycladia myrica*, which were collected from the Red Sea, Egypt, against virulent and multidrug-resistant *E. coli* isolates.

## 2. Materials and Methods

### 2.1. Sampling

Between the period of March and September 2024, a total of two hundred food samples categorized as raw milk cheese samples (n = 100) and chicken carcass samples (n = 100) were obtained from Qena city in Egypt. The raw milk cheese samples were randomly collected from different retail supermarkets, while the chicken carcass samples were collected from different retail butcher shops, representing various production batches and dates. Each sample was clearly labeled and transported in cold, aseptic containers to the laboratory of food hygiene and control, Faculty of Veterinary Medicine, Qena University, for bacteriological examination. This study was approved by the Research Ethics Committee of the Faculty of Veterinary Medicine, South Valley (Qena) University, Qena, Egypt (Code No: VM/SVU/25(9)-02).

### 2.2. Bacteriological Examination

A total of twenty-five grams of each sample was separately homogenized and enriched in 225 mL buffer peptone water (Oxoid Ltd., Basingstoke, UK) for 24 h at 37 °C with shaking. One calibrated inoculated loop (10 μL) of each sample was streaked on MacConkey agar medium (MCA: Oxoid Ltd., Basingstoke, UK). Colonies exhibiting lactose fermentation (red/pink colonies) were selected and subcultured onto Eosin Methylene Blue agar medium (EMB: Oxoid Ltd., Basingstoke, UK) and incubated overnight at 37 °C. From the subsequent growth, distinctive metallic green sheen colonies were selected and stored at −80 °C for further investigation. Only one presumptive *E. coli* colony per positive sample was selected for further analysis (i.e., one isolate per sample).

### 2.3. Biochemical Confirmation of E. coli Strains

The biochemical identification of all selected isolates at the species level was performed using the API20E system (bioMérieux SA, Marcy-l’Étoile, France). 

### 2.4. Antimicrobial Susceptibility Testing and Multiple Antibiotic Resistance (MAR) Index

Antimicrobial susceptibility testing was performed using the disk diffusion method on Mueller–Hinton (MH) agar plates (Oxoid Ltd., Basingstoke, UK), according to the Clinical and Laboratory Standards Institute (CLSI) guidelines [23]. The following antibiotic disks (Himedia, Maharashtra, India) were used: imipenem (IMP; 10 µg), meropenem (MRP; 10 µg), gentamicin (CN;30 µg), tobramycin (TOB; 10 µg), amoxicillin (AMX; 30 µg), amoxicillin/clavulanic acid (AMC; 20/10 µg), ceftazidime (CAZ; 30 µg), cefotaxime (CTX; 30 µg) and aztreonam (AT; 30 µg). Following 24 h of incubation at 37 °C, measurements of the inhibition zones were taken and interpreted.

Minimum inhibitory concentration (MIC) determination: The minimum inhibitory concentration (MIC) of colistin was determined using the broth microdilution method in cation-adjusted Mueller–Hinton broth (Bio-Rad, Marnes-la-Coquette, France). MIC values were interpreted according to EUCAST breakpoints version 16.1, 2026, with resistance defined as MIC > 2 µg/mL (https://www.eucast.org/bacteria/clinical-breakpoints-and-interpretation/clinical-breakpoint-tables/, accessed on 1 March 2026).

Multiple Antibiotic Resistance (MAR) index. The MAR index was calculated by dividing the number of antibiotics that each isolate was resistant to by the total number of antibiotics that the isolate was exposed to. The MAR index of individual *E. coli* strain from the two different sources was calculated. An MAR index value > 0.2 indicates a high-risk source of contamination where several antibiotics have been overused, and there is high selection pressure, whereas an MAR index value < 0.2 indicates a low risk source of contamination.

### 2.5. Phenotypic Detection of ESBLs

All the isolates showing resistance to either cefotaxime (CTX) or ceftazidime (CAZ) were further tested for ESBL production with the CLSI confirmatory test using both antibiotic disks of CTX (30 μg) and CAZ (30 μg) alone and in combination with clavulanic acid (CA; 10 μg). A positive ESBL phenotype is indicated by an increase of ≥5 mm in the growth-inhibitory zone around either the CTX or the CAZ disk with CA relative to the diameter around the disk containing CTX or CAZ alone. CTX-M positive *K. pneumoniae* [24] was used as a positive control and *E. coli* ATCC 25922 as a negative control.

### 2.6. Rapid ESBL NP Test for Confirmation of ESBL Production

ESBL-producing isolates were tested using the Rapid ESBL NP test [25] to confirm their resistance patterns. Briefly, one calibrated inoculated loop of the tested strain was resuspended in lysis buffer. After centrifugation, a mixture of the phenol red solution (with cefotaxime or cefotaxime and tazobactam) and the enzymatic suspension was incubated. The results were read after 30 min. A test result is considered positive when the tube containing cefotaxime alone turns from red to yellow/orange, while the well containing cefotaxime supplemented with tazobactam remains red (unchanged color).

### 2.7. Molecular Confrimation of E. coli and Genotypic Characterization of Virulence and Resistance Determinants

DNA extraction was performed using the QIAamp DNA minikit (Qiagen, Hilden, Germany), according to the manufacturer’s instructions. The extracted DNA was stored at −20 °C until further investigation. The *phoA* gene, encoding alkaline phosphatase and categorized as an *E. coli*-specific gene, was used as a molecular marker for the identification of *E. coli (*Table 1).

PCR assays were applied to detect key virulence markers in *E. coli* isolates, including Shiga toxins (Stx1 and Stx2), *eaeA* (intimin; an adhesin protein), *iss* (Increased serum survival), and *iutA* (a ferric aerobactin receptor) genes (Table 1). PCR was performed to identify ESBL- and AmpC-encoding genes (*bla*_SHV-like_, *bla*_CTX-M-like_ and *bla*_CMY-2_ genes) using the primers listed in Table 1. Then, PCR screening for *mcr-1* (plasmid-mediated colistin resistance gene) was performed. The amplification reaction was carried out using specific primers and conditions (Table 1 and Table 2). For all PCRs, 12.5 µL of 2X PCR master mix (Takara, Shiga, Japan); 1 µL of 20 pmol of each primer (Metabion, Lower Saxony, Germany) and 5 µL of template DNA were used in the reaction mixture, and nuclease-free water was added to a final volume of 25 µL. Agarose gel electrophoresis was used to visualize approximately 5 µL of each PCR product using 1.5% agarose (Sigma-Aldrich Chemie GmbH, Taufkirchen, Germany) prepared in TBE. A 100 bp DNA ladder (Qiagen, Hilden, Germany) was used to determine the fragment sizes. The gel was visualized with a UV gel documentation device (Cleaver Scientific Ltd., Warwickshire, UK). Appropriate positive, negative, and no-template controls (sterile nuclease-free water) were included in each PCR run to ensure the reliability of our results and to exclude the possibility of contamination.

### 2.8. Identification of Phylogenetic Group

The phylogenetic grouping of the 28 *E. coli* strains (A, B1, B2, D) was determined using triplex PCR [36], based on the presence or absence of *chuA, yjaA*, and *tspE4.C2* genes. The primers used in this analysis are listed in Table 1. *E. coli* strains that are negative for both *chuA* and *TSPE4.C2* are classified as group A, whereas strains positive for both *chuA* and *yjaA* fall into group B2. Strains that are negative for *chuA* but positive for TSPE4.C2 are assigned to group B1, and those that are positive for *chuA* but negative for *yjaA* are classified as group D.

### 2.9. Antimicrobial Activity of Algal Extracts Against E. coli

#### 2.9.1. Algal Collection and Preparation

Two seaweed species, *P. pavonica* and *P. myrica* (Phaeophyta), were collected from the Hurghada coast area along the Red Sea, Egypt, in March 2024, and identified as previously reported [37,38].

The fresh seaweed samples were rinsed with seawater immediately after collection to remove epiphytes, impurities, and necrotic tissues. The cleaned samples were placed in sterile polyethene bags, transported to the laboratory in an ice box, and thoroughly washed with sterile distilled water to eliminate any remaining debris. Subsequently, the samples were shade-dried at room temperature, cut into small pieces, and ground into a fine powder using a tissue grinder IKA A10 (IKA®-Werke GmbH, Staufen, Germany). The powdered samples were stored in airtight containers at room temperature until further analysis.

#### 2.9.2. Preparation of Organic Algal Extracts

Different organic solvents (ethanol, methanol, and ethyl acetate) were used for the extraction of seaweed constituents. Preliminary experiments were carried out to examine the most effective solvents for the extraction of phytochemical constituents. Ethyl acetate was determined to be the most effective organic solvent for *P. pavonica* and *P. myrica*, while methanol was also used for *P. pavonica* as the most effective organic solvent. A total of ten grams of each dried seaweed powder was extracted with 100 mL of the respective solvent under continuous stirring (150 rpm) at room temperature for 5 days. The resulting extracts were filtered through sterile Whatman No. 1 filter paper, and the filtrates were concentrated to dryness in a desiccator (Cole-Parmer Instrument Company, Chicago, IL, USA). The dried crude extracts were weighed, reconstituted in dimethyl sulfoxide (DMSO), and stored in airtight bottles under refrigerated conditions until further analysis [39].

#### 2.9.3. Gas Chromatography–Mass Spectrometry (GC-MS) Analysis of Algal Extracts

The phytochemical composition of the seaweed extracts was characterized using a TRACE 1310 GC (Thermo Fisher Scientific Inc., West Palm Beach, FL, USA) equipped with a TG-5 capillary column (30 m × 0.25 mm i.d., 0.25 μm film thickness). Analyses were performed in split injection mode using helium as the carrier gas at a constant flow rate of 1.5 mL/min. The injector temperature was maintained at 230 °C. The oven temperature was initially set at 60 °C and held for 2 min, and then increased at a rate of 10 °C/min to 300 °C, where it was maintained for 8 min. Mass spectra were acquired under electron ionization (EI) at 70 eV, with a total GC–MS run time of 35 min.

#### 2.9.4. Determination of the Minimum Inhibitory Concentration (MIC) of Algal Extracts

Bacterial isolates: A total of four MDR *E. coli* isolates were selected for testing, representing different antibiotypes, sources (chicken carcasses “CC” and raw milk cheese “RMC”), and resistance profiles (including *mcr-1*-positive and ESBL-producing isolates). The selected isolates were EC27 (CC, AB-6), EC14 (CC, AB-7), EC5 (RMC, AB-8), and EC7 (RMC, AB-9). Their detailed resistance profiles and genetic characteristics are provided in Supplementary Table S2.

Algal extract stock solution: Algal extracts were dissolved in dimethyl sulfoxide (DMSO, 1%, *v*/*v*) to obtain a 20 mg/mL stock solution, and two serial dilutions were performed in a 96-well microplate. The final concentrations of DMSO in the wells were less than 1%.

MIC determination: The MIC determination of various algal extracts against MDR *E. coli* isolates was carried out using the resazurin-based microtiter dilution method [40]. A standardized bacterial inoculum (0.5 McFarland standard) was prepared by resuspending freshly obtained (overnight) bacterial colonies. The suspension was then adjusted to obtain a working concentration of 5 × 10^5^ colony-forming units (CFU)/mL. The plate also included a positive growth control (bacteria and media without extract), and a sterility control (media and extract without bacteria). A vehicle control containing bacteria, media, and DMSO was also included to confirm that DMSO had no inhibitory effect on the tested organisms. Subsequently, the microplates were incubated at 37 °C for 24 h. To reveal bacterial growth, 30 μL of membrane-filtered resazurin dye (0.015% *w/v* in distilled water) was added after incubation. Following an additional 2 h of incubation at 37 °C, the color changes were assessed. An unchanged blue coloration indicates antibacterial activity (no bacterial growth), whereas a pink or purple coloration indicates bacterial growth and therefore a negative antibacterial activity. The MIC was determined by observing the lowest concentration of the tested algal extract at which the resazurin remained blue, indicating antibacterial activity. All experiments were conducted in triplicate on different days to ensure the accuracy and reliability of the results. Within each experiment, technical duplicates were set for each isolate. As the *E. coli* isolates used in this experiment were multidrug-resistant, including being resistant to aminoglycosides, β-lactams, and colistin, the use of an appropriate antibiotic positive control was not applicable. Instead, the validity of the assay was ensured by the consistent performance of the growth and vehicle controls included in each microplate.

### 2.10. Statistical Analysis

Statistical analyses were performed using GraphPad Prism version 9.0 (GraphPad Software, San Diego, CA, USA). The association between the number of virulence genes in each isolate and the number of resistance genes in each isolate was assessed using Pearson’s correlation coefficient (r), while the association between the phylogenetic groups identified and the presence of antimicrobial resistance genes or virulence genes were assessed using Fisher’s exact test (two-tailed). All statistical tests were two-sided, and a *p*-value < 0.05 was considered statistically significant.

## 3. Results

### 3.1. Prevalence and Molecular Confirmation of E. coli Isolated from Dairy and Poultry Samples

A total of 28 *E. coli* strains were isolated from different samples of raw milk, cheese and chicken carcass samples (n = 200). All *E. coli* isolates were biochemically identified at the species level using the API20E system (bioMérieux SA, Marcy-l’Étoile, France). The phoA gene, an *E. coli*-specific gene encoding alkaline phosphatase, was used as a molecular marker for identifying *E. coli*. Thus, all 28 isolated strains were confirmed to be *E. coli*. The overall prevalence rate of *E. coli* among the tested samples was found to be 14%, which could be, to some extent, described as a low prevalence; the prevalence was 11% (11/100) for raw milk cheese samples and 17% (17/100) for chicken carcasses samples.

### 3.2. Antibiotic Susceptibility Profile of E. coli Isolates

Ten antibiotics from various antimicrobial classes were tested against the 28 *E. coli* isolates from raw milk cheese (RMC, n = 11) and chicken carcasses (CC, n = 17) (Table 3). Interpretive criteria for disk diffusion zone diameters are provided in the footnote to Table 3. All eleven isolates recovered from RMC (100%) were found to be resistant to amoxicillin, cefotaxime, and ceftazidime; the amoxicillin/clavulanate resistance rates were high (81.8%, 9/11). Notably, all RMC isolates (100%) showed full susceptibility to imipenem, while a single isolate (9.1%, 1/11) was resistant to meropenem. High susceptibility rates were noted for the aminoglycosides gentamicin (90.9%, 1/11) and tobramycin (81.8%, 2/11). In contrast, a moderate level of resistance was observed for aztreonam (45.5%, 5/11), while a high level of resistance was found for colistin (63.6%, 7/11). In comparison, the 17 *E. coli* chicken carcass isolates showed an even higher and more extensive resistance profile to beta-lactams. All CC isolates (100%) were found to be resistant to amoxicillin and amoxicillin/clavulanate, followed by ceftazidime (94%), cefotaxime (94%), aztreonam (94%), and gentamicin (94%). Most *E. coli* chicken carcass isolates (94.1%, 16 out of 17 isolates) exhibited resistance to colistin. The MIC values of colistin ranged from 2 to 8 μg/mL. The individual colistin MIC values for all 28 isolates are provided in Supplementary Table S1. As indicated in Table 3, full susceptibility (100%) was noted against carbapenems (imipenem and meropenem), indicating excellent carbapenem activity. While the *E. coli* isolates from both sources showed a high level of resistance to β-Lactam antibiotics, the *E. coli* isolates from chicken carcasses showed a wider resistance profile across most antibiotics than those from raw milk cheese, particularly to β-Lactams, aminoglycosides, and colistin. However, imipenem and meropenem still retained excellent activity against the isolates from both sources.

**Figure 1:**
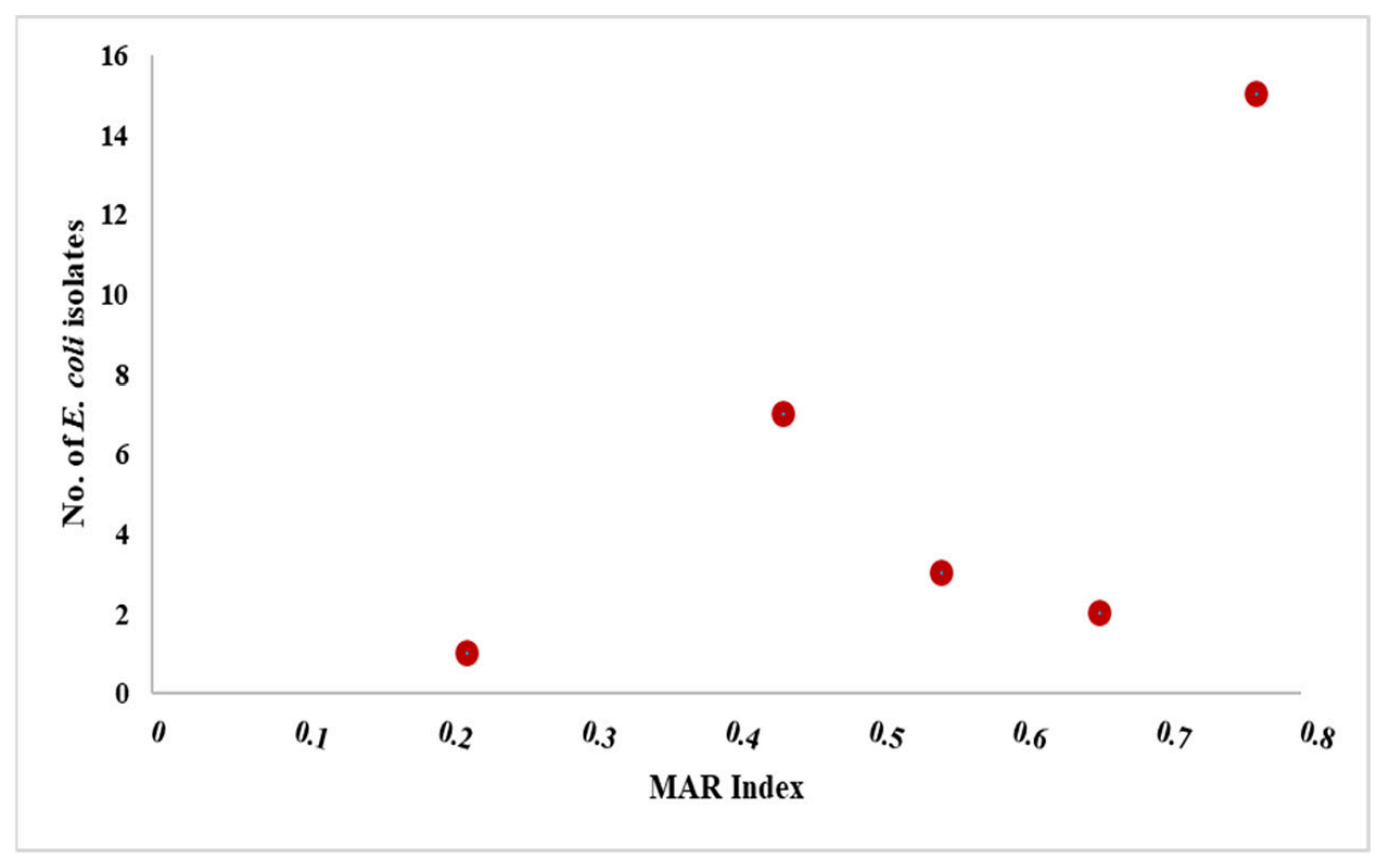
Multiple Antibiotic Resistance (MAR) index values of *E. coli* isolates isolated from raw milk cheese and chicken carcasses samples tested against different antibiotics.

The antibiogram resistance patterns and Multiple Antibiotic Resistance (MAR) indices of the *E. coli* isolates obtained from both sources are presented in Table 4 and Figure 1. Interestingly, the results showed that all 28 isolates (100%) exhibited a MAR index greater than 0.2, ranging from 0.3 to 0.8 and comprised nine distinct antibiotypes (AB-1 to AB-9) as follow; AB-1 (0.3), AB-2 (0.4), AB-3 (0.5), AB-4 (0.5), AB-5 (0.5), AB-6 (0.5), AB-7 (0.7), AB-8 (0.8), and AB-9 (0.8). Most *E. coli* isolates (n = 17, 60.7%) exhibited an MAR index of either 0.7 (n = 2) or 0.8 (n = 15), indicating widespread resistance across multiple antibiotics. It is noteworthy that, among these isolates, 15 (88.2%) were obtained from chicken carcasses and 2 from raw milk cheese samples (11.8%).

According to Magiorakos et al. [41], a multidrug-resistant (MDR) isolate is defined as an isolate that is resistant to at least one agent in three or more antimicrobial classes. Based on this definition and using 10 antimicrobial agents representing various antimicrobial classes (e.g., beta-lactams, aminoglycosides, and polymyxins), the results showed that most of the *E. coli* isolates tested were classified as MDR (64.3%, 18/28). Among these 18 MDR isolates, 16 MDR isolates (88.9%) were obtained from chicken carcasses and 2 MDR isolates (11.1%) from raw milk cheese samples (Table 4).

### 3.3. Phenotypic Screening of ESBL Production

ESBL production was evidenced in 22 isolates (78.6%) based on a phenotypic disc confirmatory test. The ESBL resistance phenotype was confirmed for those isolates by the positivity of the Rapid ESBL NP test.

### 3.4. Occurrence of Virulence Genes Among E. coli Isolates

Various virulence genes (*iss*, *iutA*, *eaeA*, *stx1*, and *stx2* genes) were identified among the *E. coli* isolates tested (Table 5). Overall, most isolates (92.9%) from raw milk cheese and chicken carcass samples were found to carry ≥3 virulence genes, indicating a high pathogenic potential. The *iss* gene was detected in all isolates (100%), while *iutA* and *eaeA* were present in the vast majority (92.9% and 89.3%, respectively). Shiga toxin genes (*stx1* or *stx2*) were less frequent, detected in 35.7% of isolates. Notably, these virulence determinants were more prevalent in the *E. coli* isolates recovered from chicken carcasses (41.2%) than in the isolates from raw milk cheese (27.3%) (Table 5).

The virulence profile (*eaeA*, *iss, iutA*/*eaeA*, and *iss*) was most frequently encountered, being present in 60.7% of the isolates and representing a virulence gene profile compatible with enteropathogenic *E. coli* (EPEC). Profiles compatible with Shiga toxin-producing *E. coli* (STEC) and enterohemorrhagic *E. coli* (EHEC) accounted for 35.7% and 28.6% of isolates, respectively (Table 5 and Table 6). Shiga toxin-encoding genes were found to be more prevalent in isolates from chicken carcasses (41.2%, 7 out of 17) compared with those from raw milk cheese (27.3%, 3 out of 11). Notably, the highly virulent Stx2-positive EHEC-compatible isolates were mainly recovered from chicken carcasses (4 out of 17, or 23.5%), with only one isolate obtained from a raw milk cheese sample. Additionally, one profile was found to be compatible with the “ExPEC pathotype” (extraintestinal pathogenic *E. coli*) in 1 out of 28 samples (3.6%).

### 3.5. Occurrence of ESBL and AmpC Producers

A high prevalence of the ESBL-encoding genes *bla*_CTX-M_ and *bla*_SHV_ was observed, being detected in 96.4% (27/28) and 85.7% (24/28) of isolates, respectively. Further, *bla*_CMY-2, a_ a plasmid-mediated AmpC gene, was detected in 17.9% of *E. coli* isolates.

### 3.6. Occurrence of MCR-1 Producers

All colistin-resistant *E. coli* isolates (23/28, 82.1%) were found to harbor *mcr-1*, with the MICs of colistin ranging from 2 to 8 μg/mL. Notably, a markedly higher prevalence rate was observed in chicken carcass isolates (16/23, 69.6%) than in the raw milk cheese isolates (7/23, 30.4%). Furthermore, co-occurrence of the ESBL gene (*bla*_CTX-M_) and the colistin resistance gene (*mcr*-1) was detected in 95.7% (22/23) of these colistin-resistant isolates.

### 3.7. Correlation Analysis Between the Virulence and Antimicrobial Resistance Genes

**Figure 2:**
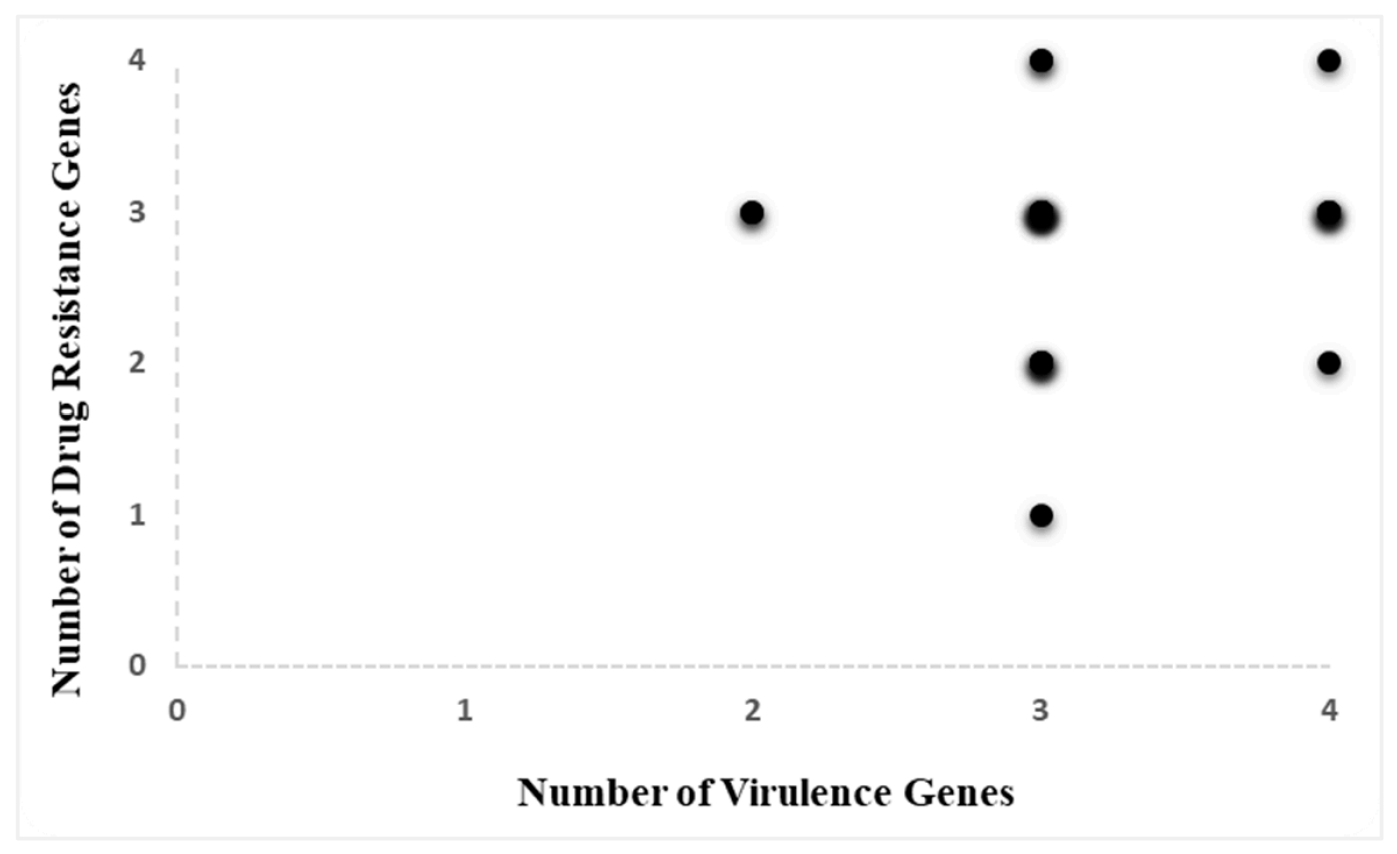
Correlation analysis between the virulence genes and drug-resistance genes identified in the 28 *E. coli* strains.

The association between the number of virulence genes in each isolate and the number of resistance genes detected was investigated using Pearson’s r and *p*-value. The results showed that the most frequent genotype encountered (46.4% of isolates) comprised *mcr-1*, *bla*_CTX-M_, *bla*_SHV_, *iutA*, *iss*, and *eaeA* (Figure 2). However, there was no statistically significant correlation between the number of antimicrobial resistance genes and the number of virulence genes in these 28 isolates (Pearson’s r = 0.09, *p* > 0.05).

### 3.8. Phylogenetic Group Identification

**Figure 3:**
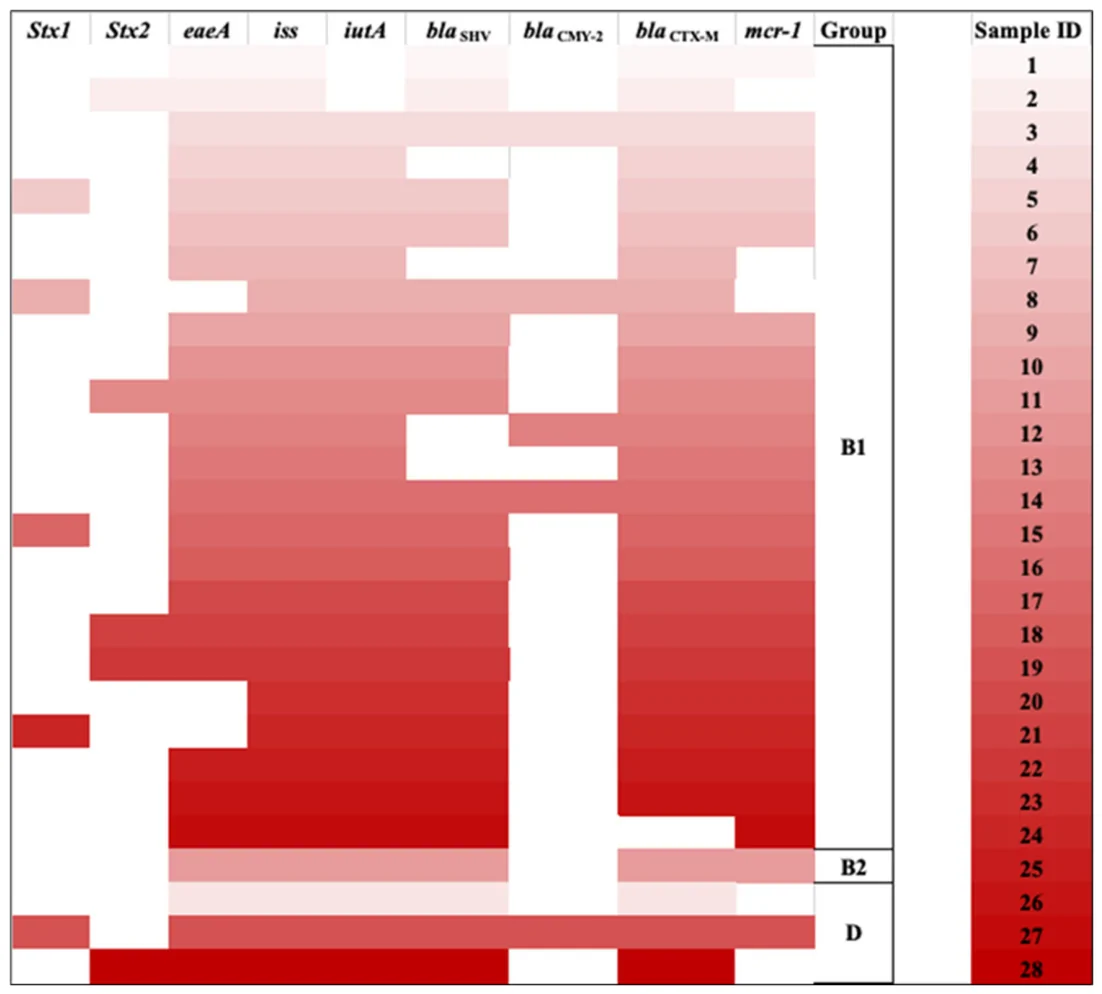
Distribution heat map showing the virulent and drug-resistant genes in the different phylogenetic groups.

The phylogenetic groups of the 28 *E. coli* strains were detected using triple PCR. The isolates from raw milk cheese were from pathogenic groups B1, B2, and D, while the isolates from chicken carcasses were mainly from pathogenic groups B1 and D (Table 7). Overall, among the 28 isolates, the majority of the strains were identified as belonging to the low pathogenic group B1 (24/28, 85.7%), and fewer strains belonged to the highly pathogenic groups D (3/28, 10.7%) and B2 (1/28, 3.5%). None of the isolates belonged to the non-pathogenic group A. The results revealed that the types of antimicrobial-resistance genes and virulence genes harbored by isolates in groups B1 and D were more diverse and abundant than those carried by group B2 isolates (Figure 3). Group B2 isolates mainly carried *eaeA*, *iss*, *iutA*, *bla*_SHV_, *bla*_CTX-M_, and *mcr-1,* but did not carry Stx1, Stx2, and *bla*_CMY-2_. Using Fisher’s exact test, no statistically significant correlation was found between phylogenetic groups (B1, B2, and D) and the presence of any antimicrobial resistance gene or virulence gene tested.

### 3.9. Antibacterial Activity of Algal Extracts Against MDR E. coli Isolates

The extracts of *P. pavonica* and *P. myrica* extracts were tested for their antibacterial activity against selected MDR *E. coli* isolates (EC5, EC7, EC14, and EC27) of different antibiotypes and sources (raw milk cheese and chicken carcasses). The methanol and ethyl acetate extracts of *P. pavonica* and the ethyl acetate extract of *P. myrica* exhibited varying levels of antimicrobial activity against the tested MDR *E. coli* isolates, with MIC values ranging from 2.5 to 10 mg/mL (Table S2). The *P. myrica* extract showed the most notable activity, with MICs of 2.5–5 mg/mL against all tested isolates. The *P. pavonica* methanol and ethyl acetate extracts showed MIC values ranging from 5 to 10 mg/mL. The vehicle control (DMSO < 1%) and growth control showed no inhibition, confirming that the antibacterial activity observed was due to the added extracts. 

### 3.10. GC-MS Analysis of the Phytochemical Constituents of Algal Extracts

**Figure 4:**
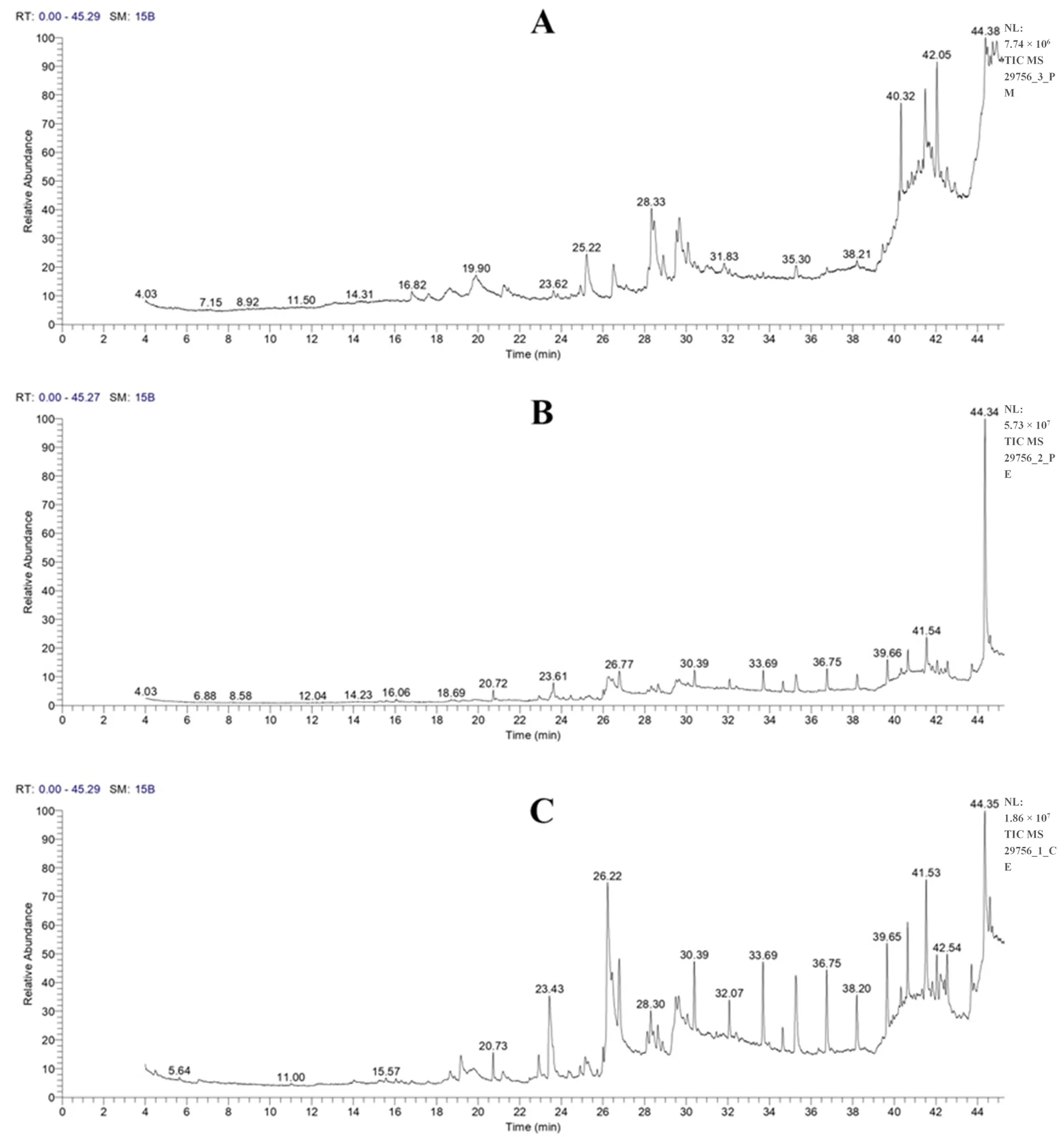
GC-MS analysis of the phytochemical constituents present in the *P. pavonica* methanol extract (**A**), *P. pavonica* ethyl acetate extract (**B**), and *P. myrica* ethyl acetate extract (**C**).

The phytochemical constituents present in the most effective crude algal extracts against the tested *E. coli* isolates were identified via GC-MS analysis (Figure 4A–C). The active compounds with their retention time (RT), molecular formula, molecular weight, and relative concentration (%) in the different algal extracts are presented in Table 8, Table 9 and Table 10. The results of the GC-MS analysis confirmed the presence of 17 compounds in the *P. pavonica* methanol extract (Table 8), nine compounds were identified in the *P. pavonica* ethyl acetate extract (Table 9), and 19 compounds in the *P. myrica* ethyl acetate extract (Table 10).

The most prevailing compounds in the *P. pavonica* methanol extract were 1-Heptatriacotanol (25.87%), 9-Octadecenoic acid (Oleic acid) (16.26%), 11-Octadecenoic acid, methyl ester (13.70%), and Cholest-5-en-3-ol (3β)- (10.26%), whereas, the major components in the *P. pavonica* ethyl acetate extract were Stigmasta-5,24(28)-dien-3-ol (3β,24Z) (38.60%), 1-Hexadecanol, 2-methyl- (24.40%), and 9-Octadecenoic acid (Oleic acid) (11.60%). Moreover, the most abundant compounds in the *P. myrica* ethyl acetate extract were 1-Hexadecanol, 2-methyl- (23.39%), 1-Heptatriacotanol (15.55%), 9-Octadecenoic acid (Oleic acid) (15.17), and Cholest-5-en-3-ol (3β)- (13.57%).

## 4. Discussion

One of the most significant reasons for the development of antibiotic resistance in humans is the widespread and uncontrolled application of nontherapeutic antibiotics in food animal production. In some countries, including the United States, approximately 80% of total consumption of medically important antibiotics “i.e., from classes important to human medicine” occurs in the animal sector, often being used in healthy livestock for non-therapeutic purposes such as growth promotion and disease prevention [42]. In this study, we detected the occurrence of extended-spectrum ß-lactamase (ESBL) producing and polymyxin-resistant *E. coli* from chicken carcasses and raw milk cheese in Egypt and determined whether the poultry meat and dairy production chain might be a reservoir and transmission route of multidrug-resistant bacteria.

Raw milk cheese, made from unpasteurized milk, has a high nutritive value, superior sensory characteristics, and textural properties compared with cheese manufactured from pasteurized milk [43]. However, it can pose health risks to consumer safety due to the potential presence of pathogens. Poultry meat has the potential to harbor and transmit foodborne pathogenic bacteria such as *E. coli* during processing, storage, and consumption. The results revealed that the prevalence rate of *E. coli* in the tested food samples can be considered relatively low (14%) compared with several previous studies. A higher prevalence of *E. coli* was found in chicken carcasses (17%) compared with raw milk cheese (11%). This higher contamination rate in poultry meat than in dairy products may be attributed to several reasons, including poultry slaughtering and processing procedures (e.g., scalding, defeathering, and evisceration) that result in cross-contamination with intestinal contents and potential bacterial survival and growth, as well as storage and transportation conditions. Similar results were obtained by the authors of [44], indicating a contamination rate of 15% in poultry and poultry-origin samples collected from a slaughterhouse in Kayseri. In agreement with our findings, Younis et al. [45] identified the presence of *E. coli* in 11.66% of chicken meat samples. Afify et al. [46] also reported that 12–18% of raw poultry meat samples from an automated poultry slaughterhouse in Egypt was *E. coli*-positive [46], while a much lower rate (4%) was reported in another study [47]. A previous study conducted by Asensi et al. [48] found that 10% of chicken carcass samples were contaminated with *E. coli*, which is slightly lower than our results. By contrast, Ibrahim et al. [49] recorded a high rate of *E. coli* contamination in raw poultry meat (40%).

The lower *E. coli* contamination rate in raw milk cheese may be multifactorial, including (i) low pH values due to fermentation, (ii) antimicrobial natural components, (iii) protective endogenous enzymes, and (iv) lactic acid production [43]. However, the growing manufacture and consumption of raw milk cheese products still poses a public health problem due to the poor hygienic conditions throughout the supply chain process. Our results are consistent with those reported in Brazil by de Campos et al., who found that in which 19% of raw milk cheeses samples were positive for *E. coli* [50]. In other studies, 14.3% of raw milk “Domiati” cheese samples examined were contaminated with *E. coli* [51], while *Escherichia coli* was found in 33% of Kareish cheese samples examined [47]. Ombarak et al. [52] reported a substantially higher contamination rates in Egyptian Kareish cheese (74.5%), while Elbastawesy et al. [51] found an overall prevalence of 26.2% in various Egyptian dairy products. Indeed, consumption of raw milk cheese might be risky for human health compared with pasteurized milk cheese. Despite the relatively low prevalence rate of *E. coli* observed in our study, the presence of *E. coli* indicates fecal contamination and inadequate hygienic practices during both cheese production and poultry slaughtering and preparations. It also highlights the potential risk of transmission of pathogenic *E. coli*, including Shiga toxin-producing *E. coli* (STEC), to consumers [53].

Even worse, the majority of the *E. coli* isolates (92.9%) from raw milk cheese and chicken carcass samples were found to be virulent and potentially pathogenic, as previously reported [51,52], highlighting the critical role played by raw milk cheese and undercooked chicken meat in the transmission of diarrheagenic *E. coli*. Notably, the *E. coli* isolates recovered from chicken carcasses were more virulent than those recovered from raw milk cheese. This may be attributed to the poultry production and slaughtering procedures throughout the farm-to-fork process, leading to consistent selection of tougher and more virulent strains. All isolates tested carried the virulence gene, *iss*. The majority of isolates also carried *iutA* and *eaeA*, followed by *stx1* or *stx2*. Approximately 60.7% of the isolates were classified as potentially enteropathogenic (EPEC) *E. coli*, a leading cause of diarrhea and premature death in children, especially in developing countries [54]. EPEC have been identified in different animal species and a variety of foods [55]. In agreement with our findings, several studies showed that EPEC was among the predominant pathotypes recovered from poultry products and raw milk cheese [52,56]. Another study reported a high proportion of contaminated chicken carcasses (39 and 56%) contaminated with EPEC at different stages of the chicken slaughtering process [57].

According to the WHO, raw milk cheese and undercooked meat products are considered common vehicles of transmission of STEC, a major foodborne pathogen worldwide [58]. Our study showed that 35.7% of the isolates were Shiga toxin-producing *E. coli* (STEC) and 28.6% were identified as potential enterohemorrhagic (EHEC) *E. coli*. STEC was isolated from 3% of raw milk cheese samples. Similar results were obtained in other studies in France (1%), Switzerland (3.7–6.3%), and Spain (1.4%) [59,60,61]. The highly virulent *stx-2*-positive EHEC isolates were mainly recovered from chicken carcasses (14.3%), except for only one isolate from a raw milk cheese sample. Several studies reported that *stx2* is more frequently linked to severe illness of hemolytic uremic syndrome (HUS) than *stx1* [62]. Fecal contamination is the most likely initial source of STEC in most foods. For instance, raw milk and, eventually, raw milk cheese are most commonly contaminated as a result of soiled udders and teats, as well as poor hygiene during milking [62]. 

Although the major animal source of STEC is cattle, poultry meat is known as a potential source of STEC contamination relative to other meat sources. In our study, 41.2% of *E. coli* isolates chicken carcass samples were identified as STEC. In another study, 38.7% of the *E. coli* isolates from raw chicken meat were characterized as STEC [63]. In addition, 15% of chicken meat cuts were contaminated with STEC in an automatic poultry dressing plant in Ismailia city, Egypt [64]. Notably, several bacterial food-borne disease outbreaks due to the consumption of undercooked or raw meat contaminated with STEC strains have been reported [65].

In our study, two *E. coli* isolates (7.1%) carried *stx1* but not *eaeA* and were therefore categorized as a potential STEC/non-EHEC hybrid (*stx1*, iss, *iutA*). While the absence of the *eae gene* excluded these *stx1*-positive strains from being classified as a “typical” Enterohemorrhagic *E. coli* (EHEC), the *eae*-negative STEC strains can still be potentially harmfull to consumer health as they can be highly virulent and cause severe diseases such as dysentery and HUS [66]. This interesting finding highlights a significant gap in the diagnosis of STEC infection. Thus, the screening for STEC infection should not rely solely on the presence of the *eae gene*. The presence of non-diarrheagenic pathogenic *E. coli*, including ExPEC, in food samples, especially in poultry products, has been reported in previous studies [67,68,69]. In this study, 3.6% of the *E. coli* isolates were classified as ExPEC.

Furthermore, the emergence of foodborne bacteria resistant to clinically important antibiotics poses a critical public health risk due to the risk of transmission of AMR determinants to human pathogens. In this study, 64.3% of the *E. coli* isolates tested were classified as MDR to beta-lactams, aminoglycosides, and polymyxins, among which 88.9% of MDR isolates were obtained from chicken carcasses. Abdelkarim et al. [70] reported that 81% of *E. coli* isolated from retail chicken meat samples in El-Sharkia, Egypt, were found to be multidrug-resistant (MDR) [70]. Unfortunately, most of these antibiotics are still considered first-line therapeutic choices for the treatment of most bacterial infections in humans, especially in developing countries. Interestingly, our study showed that the MAR index values exceeded 0.2, indicating that all isolates originated from high-risk environments. Notably, higher MAR index values were observed for chicken carcass isolates, indicating intense antimicrobial selective pressure in Egyptian poultry production systems.

In the present study, 78.6% of the *E. coli* isolates tested exhibited an ESBL-producing phenotype, with *bla*_CTX-M_ and *bla*_SHV_ representing the predominant ESBL-associated resistance genes. This high prevalence highlights the widespread dissemination of β-lactam resistance determinants among *E. coli* isolates and underscores the potential public health risks associated with their transmission through the food chain. None of the isolates were found to have any carbapenemase activity, confirming the AST results. Consistent with these findings, previous studies have documented the occurrence of ESBL-producing *E. coli* in raw milk and traditionally manufactured cheeses, highlighting dairy products as potential reservoirs of antimicrobial-resistant bacteria, in Turkey [14,71], Egypt [72], Czech Republic [73], Brazil [74], and Slovakia [15].

In our study, a total of 23 *E. coli* isolates were found to be colistin-resistant (82.1%), with MICs of colistin ranging from 2 to 8 μg/mL. All the colistin-resistant isolates were *mcr-1*-positive, with a markedly higher prevalence rate in chicken carcass isolates (69.6%) than in raw milk cheese isolates (30.4%). These results may be attributed to the use of colistin in the local farming industry in Egypt, including in poultry farms. Previous studies reported the identification of the *mcr-1* gene in *E. coli* isolates from food items such as beef sausage [20], cheese [75], and chicken meat [17]. Notably, among these 23 *mcr-1*-positive isolates, 22 isolates showed an extended-spectrum β-lactamase (ESBL) phenotype that was related to the presence of the ESBL gene (*bla*_CTX-M_). The presence of these genes corresponded closely with phenotypic resistance to β-lactams and colistin, indicating a strong association between molecular and phenotypic typing. The widespread occurrence of *mcr-1*- positive and extended-spectrum β-lactamase (ESBL)-producing *E. coli* in poultry meat and poultry farms in Egypt represents a significant public health challenge, suggesting substantial selective pressure due to the extensive use and even possible misuse of β-lactam antibiotics and colistin in animal production. The presence of these multidrug-resistant strains in poultry products raises serious concerns regarding the potential foodborne dissemination of plasmid-mediated *mcr-1* and other antimicrobial resistance determinants to humans through the handling or consumption of raw or undercooked poultry meat.

Our phylogenetic analysis revealed that phylogroup B1 was the predominant one among the isolates, regardless of the food source (raw milk cheese or chicken carcass). The identification of phylogroups D (10.7%) and B2 (3.5%) is of significant public health concern, underscoring the potential for foodborne transmission of these pathogenic phylogroups. The presence of the “*mcr-1*” colistin resistance determinant in a B2 phylogroup isolate is particularly concerning as it represents a convergence of last-resort antibiotic resistance with a phylogenetic background commonly associated with pathogenic potential [14].

In this study, we evaluated the antibacterial activity of algal extracts of two brown algal species, *P. pavonica* and *P. myrica*, against a panel of four selected MDR food isolates recovered from raw milk cheese and chicken carcasses. The MIC values obtained for the extracts ranged from 2.5 to 10 mg/mL, consistent with previously reported results [76,77,78]. Our results show that the tested extracts possess strong antibacterial activity, with the *P. myrica* ethyl acetate extract exhibiting superior activity (MICs of 2.5–5 mg/mL), compared with the *P. pavonica* extracts (MICs of 5–10 mg/mL).

The GC-MS analysis identified Oleic acid (9-Octadecenoic acid), 1-Heptatriacotanol, Cholest-5-en-3-ol (β-sitosterol/Cholesterol derivative), and 1-Hexadecanol, 2-methyl- as the most prevalent compounds across all three extracts. Oleic acid, a monounsaturated fatty acid and a major constituent found in all extracts, has strong antibacterial activity, particularly against Gram-negative bacteria, including *E. coli* [79]. Moreover, 1-Heptatriacotanol is known to exhibit antimicrobial activity by disrupting the bacterial cell membrane and increasing permeability, often acting synergistically with fatty acids [80]. Notably, the *P. myrica* extract was found to contain a branched-chain fatty alcohol, “1-Hexadecanol, 2-methyl-” (23.39%), which is known to have more potent activity as a membrane disruptor. In contrast, the *P. pavonica* ethyl acetate extract didnot have a high concentration of this component, limiting its potency and a higher concentration is required to achieve the same effect as that of the more complex *P. myrica* cocktail. In summation, the antibacterial efficacy of the *P. myrica* extract is attributed to a multifactorial synergistic effect of membrane-active fatty acids (Oleic acid), long-chain/branched alcohols (1-Hexadecanol, 2-methyl- and 1-Heptatriacotanol), and sterols (Cholest-5-en-3-ol), leading to a notable, broad-spectrum membrane-disruptive effect against MDR bacteria.

## 5. Conclusions

This study demonstrates that raw milk cheese and chicken carcasses marketed in Egypt are significant reservoirs of multidrug-resistant *Escherichia coli*, including isolates harboring extended-spectrum β-lactamase (ESBL) and *mcr-1* resistance determinants, as well as multiple virulence-associated genes. The convergence of antimicrobial resistance and virulence traits in foodborne *E. coli* underscores the potential risk posed to public health through the food chain. Importantly, the promising in vitro antimicrobial activity exhibited by the evaluated algal extracts (*P. pavonica* and *P. myrica*) against these multidrug-resistant isolates highlights their potential as natural intervention strategies for controlling such pathogens in foods. Collectively, these findings reinforce the urgent need for strengthened hygienic practices, continuous surveillance of antimicrobial-resistant bacteria, and integrated One Health approaches to mitigate the dissemination of high-risk *E. coli* strains across the food production continuum. Further studies are warranted to validate the efficacy, safety, and practical application of algal-derived antimicrobials under industrial food processing and storage conditions. Seaweeds represent a promising renewable source of natural bioactive compounds with potential applications as alternatives to synthetic antimicrobial agents. Their use may offer sustainability advantages because seaweed biomass can be obtained from marine environments without the extensive use of arable land and freshwater typically associated with terrestrial biomass production. However, the sustainability of obtaining bioactive compounds from seaweeds depends on several factors, including biomass sourcing, solvent type and consumption, extraction time, energy requirements, extraction efficiency, and waste management. Therefore, the present study considers *P. pavonica* and *P. myrica* as potentially sustainable sources of antimicrobial compounds, while recognizing that optimization of the extraction process and a comprehensive environmental assessment are necessary to establish their overall sustainability.

## Figures and Tables

**Table 1:** Oligonucleotide primer sequences used in this study.

Gene	Category	Sequence (5′ → 3′)	PCR Product	Reference
**Identification**				
***phoA***	*E. coli*-specific marker	CGATTCTGGAAATGGCAAAAG	720 bp	[26]
		CGTGATCAGCGGTGACTATGAC		
**Virulence**				
***stx1***	Shiga toxin 1	ACACTGGATGATCTCAGTGG	614 bp	[27]
		CTGAATCCCCCTCCATTATG		
***stx2***	Shiga toxin 2	CCATGACAACGGACAGCAGTT	779 bp	
		CCTGTCAACTGAGCAGCACTTTG		
***eaeA***	Intimin (adhesin)	ATG CTT AGT GCT GGT TTA GG	248 bp	[28]
		GCC TTC ATC ATT TCG CTT TC		
***iss***	Increased serum survival	ATGTTATTTTCTGCCGCTCTG	266 bp	[29]
		CTATTGTGAGCAATATACCC		
***iutA***	Ferric aerobactin receptor	GGCTGGACATGGGAACTGG	300 bp	[30]
		CGTCGGGAACGGGTAGAATCG		
**Antimicrobial Resistance**				
***bla*** **_SHV_**	β-lactamase	AGGATTGACTGCCTTTTTG	392 bp	[31]
		ATTTGCTGATTTCGCTCG		
***bla*** **_CMY-2_**	AmpC β-lactamase	TGG CCAGAACTGACAGGCAAA	462 bp	[32]
		TTTCTCCTGAACGTGGCTGGC		
***bla*** **_CTX-M_**	ESBL	ATGTGCAGYACCAGTAARGTKATGGC	593 bp	[33]
		TGGGTRAARTARGTSACCAGAAYCAGCGG		
***mcr1***	Plasmid-mediated colistin resistance	CGGTCAGTCCGTTTGTTC	308 bp	[34]
		CTTGGTCGGTCTGTAGGG		
**Phylogenetic Markers**				
***chuA***	Haem transporter	GAC GAA CCA ACG GTC AGG AT	279 bp	[35]
		TGC CGC CAG TAC CAA AGA CA		
***yjaA***	Unknown function	TGA AGT GTC AGG AGA YGC TG	211 bp	
		ATG RAG AAT GCG TTC CTC AAC		
***tspE4C2***	Unknown function	GAG TAA TGT CGG GGC ATT CA	152 bp	
		CGC GYC AAC AAA GTA TTR CG		

**Table 2:** Cycling conditions of the primers used during PCR.

Gene	Primary Denaturation	Secondary Denaturation	Annealing	Extension	No. of Cycles	Final Extension
*bla*_SHV_	94 °C/5 min	94 °C/30 s	54 °C/40 s	72 °C/40 s	35	72 °C/10 min
*bla*_CMY-2_			55 °C/40 s	72 °C/45 s		
*bla*_CTX-M_			54 °C/40 s	72 °C/45 s.		
*mcr1*			60 °C/40 s.	72 °C/40 s.		
*phoA*			55 °C/40 s	72 °C/45 s.		
*stx1, stx2*			58 °C/40 s.	72 °C/45 s.		
*eaeA*			51 °C/30 s.	72 °C/30 s.		
*iss*			54 °C/30 s.	72 °C/30 s.		
*iutA*			63 °C/30 s.	72 °C/30 s.		
*chuA*			55 °C/30 s.	72 °C/30 s.		
*yjaA*						
*tspE4C2*						

**Table 3:** Antimicrobial resistance profile of *E. coli* isolates.

Antibiotics ^a^	Antimicrobial Classes	RMC Isolates ^b^				CC Isolates ^c^			
		Resistant		Sensitive		Resistant		Sensitive	
		No.	%	No.	%	No.	%	No.	%
Amoxicillin	Aminopenicillin	11	100	0	0.00	17	100	0	0.00
Amoxicillin/clavulanate		9	81.8	2	18.2	17	100	0	0.00
Ceftazidime	3rd generation cephalosporin	11	100	0	0.00	16	94	1	6
Cefotaxime		11	100	0	0.00	16	94	1	6
Aztreonam	Monobactam	5	45.5	6	54.5	16	94	1	6
Imipenem	Carbapenem	0	0.00	11	100	0	0.00	17	100
Meropenem		1	9.1	10	90.9	0	0.00	17	100
Gentamicin	Aminoglycosides	1	9.1	10	90.9	16	94	1	6
Tobramycin		2	18.2	9	81.8	14	82.4	3	17.6
Colistin	Polymyxin	7	63.6	4	36.4	16	94.1	1	5.9

^a^ Zone diameter interpretive criteria (resistant/intermediate/susceptible, in mm) are follows: AMX: ≤13/14–16/≥17; AMC: ≤13/14–17/≥18; CAZ: ≤17/18–20/≥21; CTX: ≤22/23–25/≥26; AT: ≤17/18–20/≥21; IMP: ≤19/20–22/≥23; MRP: ≤19/20–22/≥23; CN: ≤14/15–17/≥18; TOB: ≤12/13–16/≥17. ^b^ RMC: raw milk cheese, ^c^ CC: chicken carcasses.

**Table 4:** Antimicrobial resistance patterns, multiple antibiotic resistance (MAR) indices, and MDR classification of the 28 *E. coli* isolates.

Antibiotypes	Resistance Pattern ^a^	MAR Index	Isolates ^b^			MDR Phenotype ^b^
			CC (n = 17) (%)	RMC (n = 11) (%)	Total (n = 28) (%)	
AB-1	AMX, CAZ, CTX	0.3	-	1 (9.1%)	1 (9.1%)	No
AB-2	AMX, AMC, CAZ, CTX	0.4	-	1 (9.1%)	1 (9.1%)	No
AB-3	AMX, AMC, CAZ, CTX, CST	0.5	-	4 (36.4%)	4 (36.4%)	No
AB-4	AMX, AMC, CAZ, CTX, AT	0.5	1 (5.9%)	2 (18.2%)	3 (10.7%)	No
AB-5	AMX, CAZ, CTX, AT, CST	0.5	-	1 (9.1%)	1 (9.1%)	No
AB-6	AMX, AMC, GEN, TOB, CST	0.5	1 (5.9%)	-	1 (5.9%)	Yes
AB-7	AMX, AMC, CAZ, CTX, AT, GEN, CST	0.7	2 (11.8%)	-	2 (11.8%)	Yes
AB-8	AMX, AMC, CAZ, CTX, AT, GEN, TOB, CST	0.8	13 (76.4%)	1 (9.1%)	14 (50%)	Yes
AB-9	AMX, AMC, CAZ, CTX, AT, MRP, TOB, CST	0.8	-	1 (9.1%)	1 (9.1%)	Yes

^a^ AMX: amoxicillin; AMC: amoxicillin/clavulanate; CAZ: ceftazidime; CTX: cefotaxime; AT: aztreonam; MRP: meropenem; GEN: gentamicin; TOB: tobramycin; CST: colistin. ^b^ RMC, raw milk cheese; CC, chicken carcasses; MDR, multidrug resistance.

**Table 5:** Prevalence of virulence and antimicrobial resistance genes among *E. coli* isolates from raw milk cheese and chicken carcasses.

Gene Category	Gene	Total Positive (%)	Raw Milk Cheese (n = 11)	Chicken Carcass (n = 17)
Resistance	*bla*_CTX-M_	27 (96.4%)	11 (100%)	16 (94.1%)
	*bla*_SHV_	24 (85.7%)	9 (81.8%)	15 (88.2%)
	*mcr-1*	23 (82.1%)	7 (63.6%)	16 (94.1%)
	*bla*_CMY-2_	5 (17.9%)	2 (18.2%)	3 (17.6%)
Virulence	*iss*	28 (100%)	11 (100%)	17 (100%)
	*iutA*	26 (92.9%)	10 (90.9%)	16 (94.1%)
	*eaeA*	25 (89.3%)	10 (90.9%)	15 (88.2%)
	*stx1 or stx2*	10 (35.7%)	3 (27.3%)	7 (41.2%)

**Table 6:** Virulence gene combinations and potential pathotypes of *E. coli* isolates from raw milk cheese and chicken carcasses.

Virulence Gene Combination	No. of Virulence Genes	Total Isolates (n = 28)	RMC (n = 11)	CC (n = 17)	Potential Pathotype
		No. (%)	No. (%)	No. (%)	
*stx1*, *iss*, *iutA*	3	2 (7.1%)	1 (9.1%)	1 (5.9%)	STEC/non-EHEC
*stx1*, *eaeA*, *iss, iutA*	4	3 (10.7%)	1 (9.1%)	2 (11.8%)	EHEC
*eaeA*, *iss*, *iutA*	3	16 (57.1%)	7 (63.6%)	9 (52.9%)	EPEC
*iss*, *iutA*	2	1 (3.6%)	0 (0%)	1 (5.9%)	ExPEC
*eaeA*, *iss*	2	1 (3.6%)	1 (9.1%)	0 (0%)	EPEC
*stx2*, *eaeA*, *iss*, *iutA*	4	4 (14.3%)	0 (0%)	4 (23.5%)	EHEC
*stx2*, *eaeA*, *iss*	3	1 (3.6%)	1 (9.1%)	0 (0%)	EHEC

**Table 7:** Phylogenetic group distribution of *E. coli* isolates recovered from raw milk cheese and chicken carcasses.

Group	Raw Milk Cheese	Chicken Carcass	Total (%)
A	0	0	0
B1	9	15	24 (85.7%)
B2	1	0	1 (3.5%)
D	1	2	3 (10.7%)

**Table 8:** GC-MS analysis and tentative identification of compounds present in the *Padina pavonica* methanol extract.

No.	RT	Tentatively Identified Compound	Molecular Formula	Molecular Weight	Area %
1	16.81	Caryophyllene oxide	C_15_H_24_O	220	0.71
2	17.61	Doconexent	C_22_H_32_O_2_	328	0.96
3	19.76	10,13-Octadecadiynoic acid, methyl ester	C_19_H_30_O_2_	290	0.68
4	19.91	Bergamotol, Z-à-trans-	C_15_H_24_O	220	1.10
5	24.92	Doconexent	C_22_H_32_O_2_	328	0.87
6	25.21	Hexadecanoic acid, methyl ester	C_17_H_34_O_2_	270	4.74
7	26.51	Hexadecanoic acid, ethyl ester	C_18_H_36_O_2_	284	5.91
8	28.18	6,9,12-Octadecatrienoic acid, methyl ester	C_19_H_32_O_2_	292	0.98
9	28.33	11-Octadecenoic acid, methyl ester	C_19_H_36_O_2_	296	13.70
10	29.66	9-Octadecenoic acid (Oleic acid)	C_18_H_34_O_2_	282	16.26
11	36.77	Cholestan-3-ol, 2-methylene-, (3β,5α)-	C_28_H_48_O	400	0.61
12	39.96	Aromadendrene oxide-(2)	C_15_H_24_O	220	0.76
13	40.32	2H-Pyran,2-(7-heptadecynyloxy) tetrahydro-	C_22_H_40_O_2_	336	7.40
14	41.47	5,8,11,14-Eicosatetraenoic acid, methyl ester, (all-Z)-	C_21_H_34_O_2_	318	7.93
15	42.05	Cholest-5-en-3-ol (3β)-	C_27_H_46_O	386	10.26
16	42.92	Isochiapin B	C_19_H_22_O_6_	346	1.26
17	44.37	1-Heptatriacotanol	C_37_H_76_O	536	25.87

**Table 9:** GC-MS analysis and tentative identification of compounds present in the *Padina pavonica* ethyl acetate extract.

No.	RT	Tentatively Identified Compound	Molecular Formula	Molecular Weight	Area %
1	20.72	4-t-Butyl-2-(1-methyl-2-nitroethyl) cyclohexanone	C_13_H_23_NO_3_	241	1.66
2	23.61	Neophytadiene	C_20_H_38_	278	2.92
3	26.77	9-Octadecenoic acid (Oleic acid)	C_18_H_34_O_2_	282	11.60
4	38.20	Dotriacontane	C_32_H_66_	450	2.80
5	39.65	1-Hexadecanol, 2-methyl-	C_17_H_36_O	256	24.40
6	41.53	1-Heptatriacotanol	C_37_H_76_O	536	8.99
7	42.05	Cholest-5-en-3-ol (3β)-	C_27_H_46_O	386	4.16
8	42.54	Isochiapin B	C_19_H_22_O_6_	346	4.85
9	44.34	Stigmasta-5,24(28)-dien-3-ol (3β,24Z)	C_29_H_48_O	412	38.60

**Table 10:** GC-MS analysis and tentative identification of compounds present in the *Polycladia myrica* ethyl acetate extract.

No.	RT	Tentatively Identified Compound	Molecular Formula	Molecular Weight	Area %
1	4.47	Desulphosinigrin	C_10_H_17_NO_6_S	279	0.28
2	18.66	Limonen-6-ol, pivalate	C_15_H_24_O_2_	236	0.44
3	19.16	Tributyl phosphate	C_12_H_27_O_4_P	266	1.41
4	20.73	Undec-10-ynoic acid, tetradecyl ester	C_25_H_46_O_2_	378	1.17
5	21.20	cis-5,8,11,14,17-Eicosapentaenoic acid	C_20_H_30_O_2_	302	0.53
6	23.42	1,2-Benzenedicarboxylic acid, bis (2-methylpropyl) ester	C_16_H_22_O_4_	278	4.66
7	23.61	Phthalic acid, butyl undecyl ester	C_23_H_36_O_4_	376	0.28
8	25.14	Tetradecanoic acid, 12-methyl-, methyl ester	C_16_H_32_O_2_	256	1.11
9	25.28	Alanine, 3-(benzyloxy)-, L-	C_10_H_13_NO_3_	195	1.53
10	25.73	2-Bromotetradecanoic acid	C_14_H_27_BrO_2_	306	0.32
11	26.00	cis-10-Nonadecenoic acid	C_19_H_36_O_2_	296	0.97
12	26.21	Hexadecanoic acid	C_16_H_32_O_2_	256	8.95
13	26.77	9-Octadecenoic acid (Oleic acid)	C_18_H_34_O_2_	282	15.17
14	32.42	12-Methyl-E, E-2,13-octadecadien-1-ol	C_19_H_36_O	280	0.54
15	35.26	1-Chlorooctadecane	C_18_H_37_Cl	288	5.97
16	36.75	1-Hexadecanol, 2-methyl-	C_17_H_36_O	256	23.39
17	41.53	1-Heptatriacotanol	C_37_H_76_O	536	15.55
18	43.71	Isochiapin B	C_19_H_22_O_6_	346	4.10
19	44.35	Cholest-5-en-3-ol (3β)-	C_27_H_46_O	386	13.57

## Data Availability

The original contributions presented in this study are included in the article/Supplementary Material. Further inquiries can be directed to the corresponding authors.

## Supplementary Materials

The following supporting information can be downloaded at https://www.mdpi.com/article/10.3390/foods15183324/s1, Table S1: Colistin MIC values of 28 *E. coli* isolates; Table S2: Characteristics of MDR *E. coli* isolates used for the antibacterial activity testing.
