# Supplementary material for: Multidrug-Resistant Escherichia coli Harboring ESBL and MCR-1 Genes in Raw Milk Cheese and Chicken Carcasses in Egypt: Characterization and Control Using Algal Extracts
Source: Foods. 2026 Sep 19;15(18):3324. doi: 10.3390/foods15183324 (PMC13606174; doi:10.3390/foods15183324)
Supplement: Supplementary file 1 [file foods-15-03324-s001.zip › foods-4524451-supplementary.pdf]

**Table S1**. **Colistin MIC values of 28 *E. coli* isolates.**

| Isolate ID | Source[a] | Colistin MIC (μg/mL)[b] | Interpretation (EUCAST)[c] |
|---|---|---|---|
| EC1 | RMC | 4 | R |
| EC2 | RMC | 2 | S |
| EC3 | RMC | 1 | S |
| EC4 | RMC | 8 | R |
| EC5 | RMC | 4 | R |
| EC6 | RMC | 8 | R |
| EC7 | RMC | 4 | R |
| EC8 | RMC | 2 | S |
| EC9 | RMC | 0.5 | S |
| EC10 | RMC | 4 | R |
| EC11 | RMC | 4 | R |
| EC12 | CC | 4 | R |
| EC13 | CC | 8 | R |
| EC14 | CC | 8 | R |
| EC15 | CC | 4 | R |
| EC16 | CC | 8 | R |
| EC17 | CC | 8 | R |
| EC18 | CC | 4 | R |
| EC19 | CC | 8 | R |
| EC20 | CC | 4 | R |
| EC21 | CC | 8 | R |
| EC22 | CC | 8 | R |
| EC23 | CC | 8 | R |
| EC24 | CC | 8 | R |
| EC25 | CC | 8 | R |
| EC26 | CC | 4 | R |
| EC27 | CC | 4 | R |

| | | | |
|---|---|---|---|
| EC28 | CC | 1 | S |

[a]RMC, raw milk cheese; CC, chicken carcasses
[b]Colistin MICs were determined by broth microdilution in cation-adjusted Mueller-Hinton broth. Resistance was defined as MIC > 2 μg/mL according to EUCAST breakpoints v. 16.1.
[c]R, resistant; S, susceptible.

## Table S2. Characteristics of MDR *E. coli* isolates used for the antibacterial activity testing

| Isolate ID | Source[a] | Antibiotype | Resistance profile[b] | Resistance determinants | MIC (mg/mL)[c] | | |
|---|---|---|---|---|---|---|---|
| | | | | | *P. pavonica* | | *P. myrica* |
| | | | | | Methanol extract | Ethyl acetate extract | Ethyl acetate extract |
| EC27 | CC | AB-6 | AMX, AMC, GEN, TOB, CST | *bla*$_{SHV}$, mcr-1 | 5 | 5 | 2.5 |
| EC14 | CC | AB-7 | AMX, AMC, CAZ, CTX, AT, GEN, CST | *bla*$_{CMY-2}$, *bla*$_{CTX-M}$, mcr-1 | 10 | 10 | 5 |
| EC5 | RMC | AB-8 | AMX, AMC, CAZ, CTX, AT, GEN, TOB, CST | *bla*$_{CTX-M}$, mcr-1 | 10 | 5 | 5 |
| EC7 | RMC | AB-9 | AMX, AMC, CAZ, CTX, AT, MRP, TOB, CST | *bla*$_{SHV}$, *bla*$_{CTX-M}$, mcr-1 | 10 | 10 | 5 |

[a]RMC, raw milk cheese; CC, chicken carcasses.
[b]AMX: amoxicillin; AMC: amoxicillin/clavulanate; CAZ: ceftazidime; CTX: cefotaxime; AT: aztreonam; MRP: meropenem; GEN: gentamicin; TOB: tobramycin; CST: colistin.
[c]MIC, minimum inhibitory concentration. The MIC determination was carried out using the resazurin-based microtiter dilution method. All experiments were conducted in triplicate on different days. Within each experiment, technical duplicates were also set for each isolate. The MIC values reported are the most frequent values across all replicates. DMSO (≤ 1%) served as the vehicle control and showed no inhibitory effect.
